# The Role of Microbiota in Upper Gastrointestinal Cancers

**DOI:** 10.3390/cancers17101719

**Published:** 2025-05-21

**Authors:** Giovanni Marasco, Luigi Colecchia, Daniele Salvi, Angelo Bruni, Cecilia Capelli, Elton Dajti, Maria Raffaella Barbaro, Cesare Cremon, Vincenzo Stanghellini, Giovanni Barbara

**Affiliations:** 1 IRCCS Azienda Ospedaliero Universitaria di Bologna, 40138 Bologna, Italy; giovanni.marasco4@unibo.it (G.M.); luigi.colecchia@studio.unibo.it (L.C.); angelo.bruni4@unibo.it (A.B.); cecilia.capelli3@studio.unibo.it (C.C.); elton.dajti2@unibo.it (E.D.); maria.barbaro2@unibo.it (M.R.B.); cesare.cremon@aosp.bo.it (C.C.); v.stanghellini@unibo.it (V.S.); 2 Department of Medical and Surgical Sciences, University of Bologna, Via Massarenti, 9, 40138 Bologna, Italy; 3 Department of Gastroenterology and Endoscopy, Fondazione Poliambulanza Istituto Ospedaliero, 25124 Brescia, Italy

**Keywords:** oral microbiota, gastric microbiota, Barrett’s esophagus, esophageal cancer, gastric cancer

## Abstract

The gut microbiota significantly impacts the development and progression of upper gastrointestinal (GI) cancers, including esophageal and gastric cancers. Microbial dysbiosis contributes to carcinogenesis through mechanisms such as inflammation, immune modulation, and direct DNA damage. Techniques for sampling oral, esophageal, and gastric microbiota vary, with standardization being essential for reliable results. Barrett’s esophagus (BE) and esophageal adenocarcinoma (EAC) are associated with an enrichment of Gram-negative bacteria, promoting inflammation and cancer progression. Esophageal squamous cell carcinoma (ESCC) also shows distinct microbial patterns, with reduced diversity and increased harmful bacteria like *Porphyromonas gingivalis* and *Fusobacterium nucleatum*. In gastric cancer (GC), Helicobacter pylori (HP) and non-HP gastric microbiota play significant roles, with diverse microbial communities contributing to cancer development through nitrate reduction, immune modulation, and inflammation. Emerging evidence highlights the role of non-HP bacteria in promoting carcinogenesis, with specific taxa like *Fusobacterium nucleatum* and *Lactobacillus* influencing tumor growth and immune evasion. Further research is needed to elucidate the complex interactions between gut microbiota and upper GI cancers, paving the way for novel diagnostic and therapeutic approaches. Understanding these microbial dynamics offers potential for microbiota-based interventions, improving the early detection, prognosis, and treatment of upper GI cancers. This comprehensive review summarizes the available evidence on the role of microbiota in upper GI oncology and the need for continued exploration in this field.

## Simple Summary

The gut microbiota, the bacteria, fungi, and virus residing in our gastrointestinal tract, plays a key role in the development of upper gastrointestinal cancers, such as esophageal and gastric cancers. Changes in the microbial balance (dysbiosis) can promote cancer through inflammation, immune changes, and DNA damage. In esophageal cancer, potentially harmful bacteria are more common in precancerous conditions. In gastric cancer, it is known that *Helicobacter pylori* is a potent carcinogen, but a new field of study has shown that other gastric bacteria can contribute to cancer progression. Understanding these microbial changes could lead to better ways to diagnose patients and, eventually, treat or prevent cancer.

## 1. Introduction

Upper gastrointestinal cancers, including esophageal and gastric cancers, represent significant global health challenges due to their high incidence and mortality rates [1]. The human gastrointestinal tract is home to a diverse and dynamic microbiota, which has been increasingly recognized for its role in health and disease [2]. Digestive tract microbiota refers to the microorganisms inhabiting the human digestive tract, including the oral, gastric, esophageal, and intestinal microbiota. It is established at birth and further shaped during breastfeeding, becoming relatively stable by late childhood and continuing through adolescence into adulthood [2]. The esophagus, typically containing about 10^4^ bacteria per gram of content, predominantly hosts Gram-positive bacteria, such as *Streptococcus*, *Prevotella*, and *Lactobacillus*, with less abundant genera like *Peptostreptococcus*, *Neisseria*, and *Actinobacillus* [3]. Dysbiosis, or microbial imbalance, in the esophagus can result from various factors, including diet, chemical exposure, alcohol, infections, or medications, leading to altered immune responses and chronic inflammation that may contribute to cancer development [4]. Esophageal cancer (EC) primarily comprises esophageal squamous cell carcinoma (ESCC) and esophageal adenocarcinoma (EAC), with ESCC being more prevalent in eastern countries and EAC in western countries [5]. EAC often develops from Barrett’s esophagus (BE), a condition characterized by the replacement of the normal squamous epithelium with columnar metaplasia due to chronic gastroesophageal reflux disease (GERD) [6,7,8]. The progression from GERD to BE and subsequently to EAC is linked to significant alterations in the local microbiota, with an enrichment of Gram-negative bacteria such as *Fusobacteria*, *Enterobacteriaceae*, *Sphingomonas*, *Proteobacteria*, and *Campylobacter* [9,10]. These bacteria can release lipopolysaccharides (LPSs) that activate inflammatory pathways, promoting cancer progression [11]. In gastric cancer (GC), the role of *Helicobacter pylori* (HP) is well established, but emerging evidence highlights the importance of non-HP gastric microbiota in carcinogenesis [12]. The gastric microbiota, influenced by intrinsic factors like low pH and antimicrobial enzymes and extrinsic factors such as diet and antibiotic use, undergoes significant changes during the progression from chronic gastritis to GC [13,14]. Also, nitrate-reducing bacteria, such as *Escherichia*, *Shigella*, and *Lactobacillus*, can increase concentrations of carcinogenic nitrite and N-nitroso compounds in the stomach [15,16]. Additionally, bacteria like *Fusobacterium nucleatum* can induce inflammatory responses and modulate the host immune system to favor tumor growth [17,18,19].

This review aims to provide a comprehensive overview of the current understanding of the gut microbiota’s role in upper GI cancers, exploring the mechanisms of microbial dysbiosis in carcinogenesis, potential biomarkers for early detection and prognosis, and emerging strategies for microbiota modulation as therapeutic interventions.

## 2. Sampling of Oral, Esophageal, and Gastric Microbiota: Techniques and Relevance

### 2.1. Oral Microbiota Sampling

The oral microbiota exhibits significant heterogeneity depending on the specific site sampled. Different regions of the oral cavity harbor distinct microbial niches. Before even selecting different techniques for sampling the oral microbiota, a challenge arises between using stimulated versus unstimulated saliva sampling methods. Stimulated salivation may cause dilution and introduce bias into the results. On the other hand, unstimulated saliva, collected after several mouth rinses, may result in a low total volume, limiting its use in multi-omics analyses [20]. In a well-designed study, Roca et al. compared the microbiome composition in patients using both stimulated and unstimulated saliva, showing notable differences within the same participants. This study demonstrated that stimulated saliva provided a more comprehensive representation of the oral microbiota [20].

**Figure 1:**
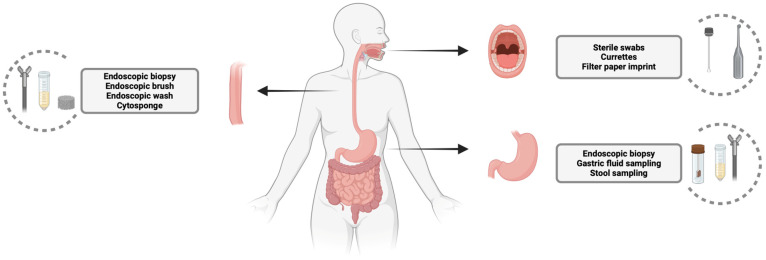
Main diagnostic tests for analyzing microbiota across different sections of the upper gastrointestinal tract.

The most common sampling techniques for oral microbiota analysis involve the use of sterile swabs for regions like the tongue dorsum, hard palate, palatine tonsils, and oropharynx, while sterile curettes are used for collecting supra- and subgingival plaques (Figure 1) [21]. These methods have been proposed as part of a standard protocol for oral cavity sampling and have been utilized to define the oral microbiome in healthy individuals using whole-genome sequencing (WGS) [22,23]. An innovative technique using a filter paper imprint has also been validated by comparing it with the swab method, showing comparable results for buccal mucosa sampling. The filter paper method offers advantages, such as calculating the microbial colonization density over a defined unit area and the improved analysis of Gram-negative bacteria due to its suction mechanism [24]. However, the validation study relied on a culture-based microbiota analysis rather than the more advanced next-generation sequencing techniques now commonly used. Therefore, the standardization of sampling techniques is essential to ensure reliable and reproducible testing results across studies and clinical applications.

### 2.2. Esophageal Microbiota Sampling

Esophageal microbiota sampling methods can be categorized into invasive and non-invasive techniques (Figure 1). Invasive methods, which require an endoscopy, are currently the gold standard for microbiota sampling and are often used as a comparator for newer techniques [25,26,27,28,29,30]. Endoscopic biopsies allow for the analysis of a mucosa-associated microbiome which is in direct contact with the mucosal cells. However, this method has several limitations, including the analysis of only a small tissue section (3–5 mm), being time-consuming, and carrying the risk of potential complications. Like biopsies, brushes are sometimes used to sample a wider surface area. However, it has been suggested that brushes mainly collect firmly attached microbes from the mucosal surface, potentially missing loosely attached microorganisms [25]. Additionally, the mechanical forces involved in brushing may alter the microbiota composition.

To target the loosely attached microbes, a non-invasive technique, though still requiring an endoscopy, has been introduced: endoscopic wash sampling [27]. A Japanese study found that in patients with esophageal cancer, the alpha diversity measures (the Shannon Index, Observed Features, and Simpson Index of Evenness) in esophageal wash samples significantly correlated with the microbiota from normal esophageal mucosal biopsies but not with the microbiota from cancerous tissue, suggesting that the wash method may be useful for analyzing normal esophageal microbiota [29]. However, an endoscopy is still required, and the risk of contamination from the oral microbiota remains.

A non-invasive cell-sampling device, the Cytosponge, has also been proposed. It is a safe and well-tolerated method in which a spherical mesh attached to a string is swallowed, expands in the stomach, and is then withdrawn, collecting cells from the esophagus [31]. In a case–control study, Elliott et al. demonstrated that the Cytosponge yielded more than ten times the microbial DNA compared to endoscopic brushes or biopsies, as measured by a quantitative PCR [30]. This could be due to its ability to sample a larger portion of the esophagus, although contamination from the stomach or oral cavity is still a potential concern. Similar findings have been confirmed in a more recent comparative study between the Cytosponge and brush sampling techniques [25]. A similar device, the esophageal string test, has been proposed for use in the pediatric population. It has demonstrated comparable microbial compositions to those found in matched esophageal biopsies, suggesting it could serve as a promising non-invasive method for studying the esophageal microbiome in children [28].

### 2.3. Gastric Microbiota Sampling

The two most used methods for sampling the gastric microbiota are biopsy and stool sampling (Figure 1). Similarly to the approach for esophageal sampling, biopsies involve an invasive procedure and are limited to specific target regions.

In contrast, stool sampling is a non-invasive, patient-friendly method that is cost-effective and yields sufficient biomass for the microbial analysis [32]. However, stool samples predominantly represent microbial communities from the terminal colon rather than the entire gut, providing a limited perspective. It has been demonstrated that the microbiota composition, viral activity, host proteome, and bile acid profiles vary substantially along the GI tract, affecting the accuracy of stool samples in capturing the full longitudinal and temporal variability of the microbiota [33].

An alternative approach to characterizing the gastric microbiota involves sampling gastric fluid, typically obtained during an endoscopy [34,35,36]. However, limitations of gastric juice sampling have been noted, including the potential detection of bystander microbes, the risk of contamination from endoscopic sampling methods, and a low bacterial load. To reduce the invasiveness, a novel gastric fluid sampling device utilizing a disintegrating polymeric blocking disk has been proposed [37]. Initial in vitro, in situ, and ex vivo animal studies demonstrated the device’s safety and accuracy, though no human models have been tested.

Additionally, Park et al. developed a capsule robot designed for sequential sampling at six sites within the gastrointestinal tract, including the stomach [38]. This device minimizes the cross-contamination between samples and is navigated via an electromagnet system, equipped with a camera to document sampling locations. To date, only ex vivo and in vivo tests on porcine models have been conducted, highlighting the need for further research to evaluate its efficacy in humans.

## 3. Microbiota in Esophageal Cancer

Esophageal cancer (EC) is a significant global health challenge, ranking as the eighth leading cause of cancer-related deaths worldwide [5]. EC mainly comprises esophageal squamous cell carcinoma (ESCC) and esophageal adenocarcinoma (EAC), with ESCC being predominant in eastern countries and EAC in western countries [5]. EAC typically develops from Barrett’s esophagus (BE) at the distal esophagus, while ESCC can initiate in any part of the esophagus usually due to the direct exposure to carcinogens, such as chemicals, cigarette smoke, and alcohol [1]. Symptoms of EC are closely linked to disease progression. In the initial stages, patients may be asymptomatic or suffer from mild dysphagia, while advanced stages show worsening dysphagia with persistent chest or back pain and considerable weight loss [1]. As far as therapeutic management is concerned, for early-stage disease, endoscopic therapy is a common approach for removing the tumor tissue infiltrating the mucosa or submucosa while chemotherapy and radiation therapy are performed either before or after a surgical resection for locally advanced disease [39]. For metastatic disease, instead, treatment options include chemotherapy, radiation, targeted therapy, immunotherapy, or their combination [39].

Upper digestive microbiota has been hypothesized as a contributing factor in EC development and progression. Typically, the esophagus contains about 10^4^ bacteria per gram of content, mainly Gram-positives [40]. The most common identified bacteria include *Streptococcus*, *Prevotella*, and *Lactobacillus*, with *Peptostreptococcus*, *Neisseria*, and *Actinobacillus* being less abundant and rarely reported [3]. Furthermore, a relatively low abundance of viruses, such a Betaherpesvirus and Papillomavirus, and fungi, such as *Candida glabrata*, have been found [41]. Although most studies on esophageal microbial communities have been conducted on the lower esophageal mucosa, it has been shown that the microbiota composition is also similar in the proximal and medial esophagus, with a substantial overlap with the microbial composition of the mouth, pharynx, and stomach [42,43]. The esophageal microbiota can be altered by diet, chemical exposure, alcohol, infections, or medications that disrupt the normal balance between bacterial geni and between microbiota and the esophageal immune system. This microbial imbalance leads to an altered immune response and the chronic inflammation of the esophageal mucosa, which may contribute to the development and progression of cancer [4].

### 3.1. Microbial Alterations in Barrett’s Esophagus

Barrett’s esophagus is a condition in which columnar metaplasia replaces the normal squamous cell epithelium of the esophagus. The major risk factor for BE is gastroesophageal reflux disease (GERD). A chronic exposure to stomach acid may cause the inflammation of the esophagus and initiate the cellular changes that characterize this disease [6,7,8]. The exact molecular mechanism for this shift is not yet known; however, it was demonstrated that the chronic exposure of the esophageal mucosa to gastric acid results in increased intercellular spaces which allow hydrochloric acid molecules to permeate down to the stem cells in the basal layer, modifying their differentiation [6,7,8]. GERD, BE, and EAC define a sequence that begins with GERD and progresses with BE development, followed by dysplasia (first low-grade and then high-grade), and finally EAC development [8].

Recent data have emerged on the local gastroesophageal microbiota’s composition being altered in neoplastic and pre-neoplastic lesions and playing a crucial role in the pathological process from BE to adenocarcinoma. The first observed key feature of the esophageal microbiota in GERD and BE is a relative enrichment of Gram-negative bacteria, particularly *Fusobacteria*, *Enterobacteriaceae*, *Sphingomonas*, *Proteobacteria*, and *Campylobacter* [9,10]. Subsequent studies comparing BE and healthy controls confirmed the Gram-negative shift but showed fewer taxonomic differences, such as increased relative abundances of *Leptotrichia* [44,45], *Capnocytophaga*, *Gemella*, *Veillonella*, and *Streptococcus sanguinis* [45]. Although these studies showed interesting results and the selection criteria were stringent, it should be noted that the population assessed was rather small. Regarding further differences in the characteristics of BE, a prospective study assessed the microbial population of 74 patients, including 34 patients with BE and 40 patients without BE, finding that as Barrett’s length increased, multiple organisms, such as *Corynebacterium*, *Dialister*, *Gemella*, *Haemophilus*, *Leptotrichia*, *Neisseria*, *Prevotella*, *Rothia*, *Streptococcus*, and *Veillonella*, were less likely to be detected [46]. A possible pathophysiological mechanism linking Gram-negative bacteria to the esophageal adenocarcinoma cascade involves lipopolysaccharides (LPSs). In fact, LPSs released by Gram-negative bacteria can activate the NF-κB pathway, leading to IL-8 expression in immune cells, which in turn activates toll-like receptors (TLRs) and nucleotide-binding oligomeric domain (NOD)-like receptors, triggering chronic inflammation and facilitating cancer progression [11]. Similarly, higher levels of the proinflammatory cytokine IL-18 were found in subjects colonized by *Campylobacter*, a Gram-negative bacterium, in the context of BE [47]. Furthermore, Gram-negative bacteria-derived LPSs may relax the lower esophageal sphincter and postpone gastric emptying, contributing to the increase in acid reflux and the molecular changes in the esophageal epithelium caused by acid reflux [48].

**Figure 2:**
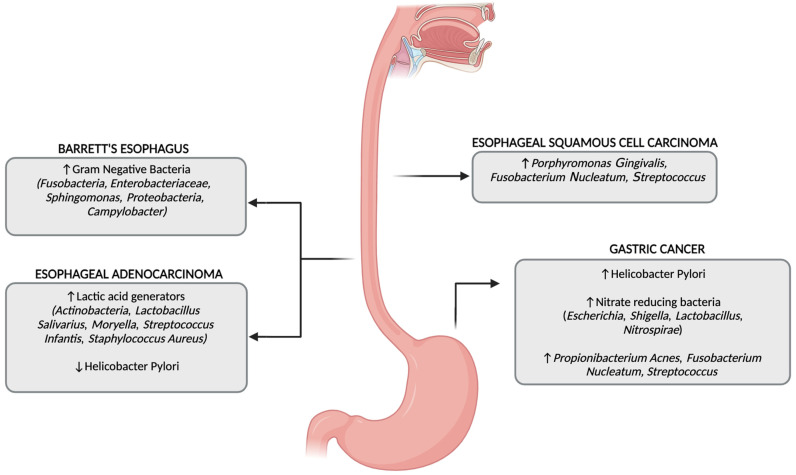
Main microbial alterations found in neoplastic and pre-neoplastic conditions of the upper gastrointestinal tract; ↑, increase; ↓, decrease.

Finally, Gram-negative bacteria, such as *Escherichia coli* and Campylobacter, directly induce DNA damage via the production of alkylating agents as well as reactive oxidative species, promoting cancer development [49]. The role of HP is still debated, although various recent meta-analyses [50,51] have shown a reduced rate of EAC in patients colonized by HP. The main microbial alterations found in Barrett’s esophagus and esophageal cancers are summarized in Figure 2.

### 3.2. Microbial Alterations in Esophageal Cancer

In the setting of EAC, the esophageal microbiota is distinct from that of the healthy esophagus. *Actinobacteria*, *Lactobacillus salivarius*, *Moryella*, *Streptococcus infantis*, and *Staphylococcus aureus* have all been reported to be more represented in this population [52]. A fundamental shared trait of these bacteria is lactic acid generation. Long-term elevated lactate levels, in addition to the pathophysiological changes brought by Gram-negatives, have been shown to enhance carcinogenesis and progression by promoting angiogenesis, immunological evasion, cell migration, and metastasis [52]. Moreover, a sustained acidic environment inhibits the growth of other potentially beneficial bacteria, creating a self-sustained harmful environment [53]. In a pivotal case–control study, oral microbiome samples from 17 control patients, 16 patients with BE without dysplasia, 6 with low-grade dysplasia (LGD), 5 with high-grade dysplasia (HGD), and 5 with esophageal adenocarcinoma (EAC) have shown that the most significant change in the microbiome happened between the stages of LGD and HGD [11]. In fact, when compared to the group of patients with BE or LGD, patients with HGD or EAC exhibited a substantial decrease in the Firmicutes phylum and an increase in Enterobacteriaceae, a family of Gram-negative bacteria. Additionally, a lower microbial alpha-diversity, a measurement of the local bacterial variability, was found in EAC compared with BE [11]. In this study, the recent use of antibiotics was considered as an exclusion criterion; however, other significant confounding factors, such as the type of diet, use of proton pump inhibitors, or oral hygiene status, were not taken into consideration.

Regarding ESCC, a case–control study on 18 patients with ESCC and 11 controls has shown a reduction in microbial diversity with respect to healthy controls, with increased *Porphyromonas* (*P*.) *gingivalis* and *Fusobacterium nucleatum* [54]. Although limited by the small sample size, this study confirmed the hypothesized role of *Porphyromonas gingivalis*, a Gram-negative bacterium linked to periodontal disease, as capable of promoting ESCC progression by activating the NF-κB and IL-6/STAT3 pathways. Studies show *P. gingivalis* enhances tumor growth, chemotherapy resistance, and is associated with a poor prognosis in ESCC patients [54]. Additionally, *Fusobacterium nucleatum* can stimulate chemokines, such as CCL20, known to aid tumor invasion [52].

### 3.3. Microbiota and Esophageal Cancer Screening and Prognosis

Identifying precise biomarkers for early BE or EC detection is crucial to improve clinical outcomes. The standard of care for people with non-dysplastic BE includes an upper endoscopy every three years to appropriately screen for the development of EAC [8]. This method is limited by the need for repeated invasive procedures, the associated healthcare costs, and the unclear benefits in terms of the lower EAC incidence or mortality [55]. Non-invasive techniques, such as a microbiota analysis via oral swabs and brushings from the distal esophagus, have been tested in the previous years.

In the context of EAC, for example, a model using the relative abundance of *Lautropia*, *Streptococcus*, and *Bacteroidales* in the saliva was developed to distinguish BE from controls with a high accuracy (AUROC 0.94, 95% confidence interval (CI): 0.85–1.00) [11]. Likewise, in ESCC, other models including *Actinomyces*, *Fusobacterium* (*F.*) *nucleatum*, *Haemophilus haemolyticus*, *Porphyromonas gingivalis*, and *Streptococcus australis* extracted by a salivary swab have been proven to be feasible to detect early ESCC in a total of 449 specimens collected from 349 participants with different esophageal diseases (AUROC 0.722, 95% CI: 0.621–0.823) [56]. Although promising, these tests are still limited by the low number of enrolled patients, the availability of tests, and the need for validation.

Microbiota show potential not only for diagnosing EC but also as prognostic markers. Among microbial candidates, *F. nucleatum* seems to be the most promising. High *F. nucleatum* levels are correlated to a larger tumor size, advanced EC stages, and a worse prognosis, particularly in recurrent cases [57]. Similarly, *Porphyromonas gingivalis* was detected more frequently in the oral microbiome of patients with ESCC than in healthy controls (57% vs. 24% *p* = 0.0034) and was associated with advanced clinical stages and a worse prognosis (Hazard Ratio = 2.14, *p* = 0.014) [58]. Moreover, the esophageal microbiota sampling in lower esophageal biopsies showed an increased *Streptococcus* abundance in patients with advanced ESCC stages (T3/T4) with respect to more confined disease (T1/T2) [59]. These findings highlight the importance of the gut microbiota in early EC detection and prognosis, with further clinical trials needed to translate these findings into clinical practice.

### 3.4. Microbiota Modulation in Esophageal Cancer

A new field of study that shows potential for enhancing preventative and therapeutic approaches is microbiota manipulation in esophageal cancer. Proton pump inhibitors (PPIs) are widely used medications to treat GERD and are known to actively alter the gut microbiota [60]. In a case–control study conducted in the United States on 45 patients with BE it was found that after an 8-week course of PPIs, PPI users exhibited higher relative abundances of *Streptococcus* and lower relative abundances of Gram-negative bacteria as compared to controls, which are known to be markers of neoplastic progression. However, these results should be interpreted with caution since the PPI users were a mix of BE and non-BE patients [61]. Although direct studies are lacking, it could be speculated that PPI therapy may help patients with precancerous esophageal lesions by reversing the Gram-negative shift seen in the BE to EAC cascade [62]. More data are available on the role of dental hygiene in EC. A recent analysis on 163 patients who underwent surgery with perioperative oral care showed that poor oral hygiene, evaluated via radiological measures and classified by dentists into three groups of severity, was a significant independent prognostic factor for poor overall survival, making oral hygiene a simple and affordable preventive target for EC [63]. Finally, targeted dietary interventions may be used as new EC preventive strategies. For example, a meta-analysis encompassing 15 studies involving 16,885 subjects, showed that subjects with higher daily fiber intakes had a decreased risk of EAC (for an increment of 10 g/day, RR = 0.79, 95% CI: 0.69–0.92), although this was limited by the lack of specific daily fiber quantities [64]. In fact, the effect of fiber is linked to its ability to reduce the relative abundance of esophageal Gram-negative bacteria which contribute to the adenocarcinoma cascade [64,65].

In conclusion, esophageal cancer is a significant global health issue. Factors such as diet and chemical exposure may disturb the healthy esophageal microbiota, leading to dysbiosis and, hence, chronic inflammation and neoplastic changes. Indeed, several unique microbial communities have been defined in the healthy esophagus, BE, and progressive stages of esophageal adenocarcinoma. Although an increase in Gram-negative bacteria is commonly observed, the precise determination of the causal role of microbiota in cancer formation is still lacking. In esophageal squamous cell carcinoma, there is a decrease in microbial diversity and an increase in the harmful bacteria, such as *Porphyromonas gingivalis*, associated with lower overall survival. Although the available evidence remains scant, mainly due to the limited sample size evaluated and the missing evaluation of several confounding factors, identifying changes in the microbiota and potentially modifying it could help the early detection and treatment of esophageal cancer.

## 4. Microbiota in Gastric Cancer

### 4.1. Microbiota in Gastric Cancer

The human stomach, historically considered a sterile environment due to its acidic conditions and digestive enzymes, is now acknowledged to harbor a diverse and dynamic microbiota. Advances in high-throughput sequencing and microbial culture techniques have unveiled a complex community of microorganisms residing in the gastric mucosa and lumen [66]. While *Helicobacter pylori* (*HP*) is a well-established pathogen linked to GC and mucosal-associated lymphoid tissue (MALT) lymphoma, emerging evidence highlights the significant role of non-HP gastric microbiota in gastric carcinogenesis [12]. Not enough data are available on the role of non-HP gastric microbiota in MALT-lymphoma; therefore, we aimed to summarize the available data on gastric adenocarcinoma.

Early studies utilizing the temperature gradient gel electrophoresis of 16S rRNA amplicons identified three predominant bacterial phyla in the stomach: *Proteobacteria*, *Firmicutes*, and *Actinobacteria* [67,68]. Subsequent research employing G2 Phylo-Chip and 16S sequencing expanded this composition, revealing up to 44 bacterial phyla, with *Actinobacteria*, *Firmicutes*, *Bacteroidetes*, *Proteobacteria*, and *Fusobacteria* being dominant [69]. Common genera include *Streptococcus* (Firmicutes), *Neisseria* and *Haemophilus* (Proteobacteria), and *Prevotella* and *Porphyromonas* (Bacteroidetes) [70].

The gastric microbiota is influenced by intrinsic factors such as a low pH, antimicrobial enzymes like pepsin, immunoglobulins (e.g., IgA), and antimicrobial peptides [71]. Extrinsic factors include diet, antibiotic use, proton pump inhibitors (PPIs), geographic location, and surgical interventions [13,14]. Long-term PPI use elevates the gastric pH, facilitating bacterial overgrowth and translocation, particularly of oral bacteria like *Peptostreptococcus stomatis* and *Streptococcus anginosus* [72]. Surgical procedures such as subtotal gastrectomy alter the gastric environment, promoting the growth of bacteria such as *Prevotella* and *Streptococcus* [73]. Moreover, recent investigations have demonstrated that patients with GC often display a marked decrease in the overall bacterial diversity in their gastric microbiota, with a significant enrichment of genera such as *Clostridium* and *Fusobacterium*, as reported by Hsieh et al. [74].

This dysbiosis pattern can vary according to factors like the geographic location and the presence of *H. pylori*, highlighting the multifaceted interactions within the gastric ecosystem.

### 4.2. Non-Helicobacter Pylori Microbiota Alteration in Gastric Cancer

The composition and functionality of the gastric microbiota undergo significant changes during the progression from chronic gastritis to GC, a process often described by the Correa cascade. This progression is accompanied by a state of microbial dysbiosis, which is characterized by a reduced overall diversity but an increased abundance of specific pathogenic taxa in the gastric mucosa [66,75].

Microbiota imbalance is not merely a secondary effect of the gastric pathology but actively contributes to carcinogenesis through several mechanisms. Nitrate-reducing bacteria can increase the concentrations of nitrite and N-nitroso compounds in the stomach, which are potent carcinogens [15]. As a matter of fact, Ferreira et al. retrospectively evaluated the differences in the gastric microbiota of 54 patients with gastric carcinoma and 81 patients with chronic gastritis by 16S rRNA gene profiling, using next-generation sequencing, finding a significant increase in nitrate reductase and nitrite reductase activities, promoting the generation of nitrite and nitric oxide, leading to DNA damage [67]. *Escherichia*, *Shigella*, *Lactobacillus*, and *Nitrospirae*, known for nitrate/nitrite metabolism, are enriched in GC tissues [16].

Moreover, non-HP bacteria can induce inflammatory responses by activating immune cells and signaling pathways. *Propionibacterium acnes* has been recently identified as a strong risk factor for GC development [76]. It activates the natural killer group 2 member D (NKG2D) system and upregulates proinflammatory cytokines, like interleukin-15, leading to persistent inflammation and promoting carcinogenesis [71,77]. *F. nucleatum*, commonly implicated in colorectal cancer, has also been detected in gastric cancer, where it fosters a proinflammatory microenvironment conducive to tumor progression and modulates the host immune response in favor of oncogenesis [18]. In fact, *F. nucleatum* produces adhesins such as Fap2, which bind to immune receptors on natural killer (NK) and T cells, inhibiting their cytotoxic activity and allowing the immune evasion of cancer cells [19]. Another adhesin secreted by *F. nucleatum*, FadA, can instead bind to E-cadherin, thereby activating the Wnt/β-catenin pathway and driving malignant transformation [74,78]. Moreover, *F. nucleatum* releases endotoxins that can suppress immune responses, upregulate transcription factors such as NF-κB, and stimulate the secretion of proinflammatory cytokines (IL-1β, IL-6, IL-8, TNF), which lead to an oncogenic environment [79,80]. For these reasons, higher relative abundances of *F. nucleatum* have been linked to an advanced disease stage and poorer prognosis [81]. Finally, *Propionibacterium acnes* and *F. nucleatum*, besides inducing inflammation and immune evasion, can directly trigger DNA damage responses in host cells, contributing to genomic instability and promoting cellular proliferation and invasiveness [81,82].

In the context of advanced gastric lesions in mice models, it has been demonstrated that the gastric colonization by a restricted consortium of commensal bacteria—specifically *Clostridium* sp. *ASF356*, *Lactobacillus murinus ASF361*, and *Bacteroides* sp. *ASF519*—induces gastritis, atrophy, and dysplasia to a degree comparable to that driven by a more diverse microbiota, independently of Helicobacter pylori infections [83]. This suggests that even commensal bacteria can promote gastric carcinogenesis in the context of gastric inflammation and atrophy. For instance, *Lactobacillus* species, while often considered beneficial, have been found to be enriched in GC patients and may influence the gastric environment to favor carcinogenesis [77]. In a meta-analysis involving 1642 gastric biopsy samples and 394 stool samples, researchers found that *Lactobacillus* and *Streptococcus* were consistently enriched in GC patients [84]. Their abundance was associated with the progression of gastric carcinogenesis, and their combination in diagnostic models showed a high accuracy and reproducibility across different cohorts. These events promote a persistent inflammatory milieu and foster tumor cell proliferation, invasion, and survival. The main microbial alterations found in gastric cancer are summarized in Figure 2.

### 4.3. Microbiota and Gastric Cancer Screening and Prognosis

Mounting evidence indicates that alterations in the gastric microbiota may serve as valuable biomarkers for the early detection of GC and inform the development of novel therapeutic strategies. Indeed, multiple studies have identified distinct microbial signatures in patients with GC compared to those with non-malignant gastric conditions, underscoring the potential for these profiles to refine diagnostic accuracy and guide personalized interventions. Huang et al. aimed to characterize the salivary microbiota in patients at different progressive histological stages of gastric carcinogenesis in order to identify relevant microbial markers for gastric cancer detection in 293 patients with superficial gastritis [number (*n*)= 101], atrophic gastritis (*n* = 93), and gastric cancer (*n* = 99). A distinct salivary microbiota in patients with GC compared to superficial and atrophic gastritis was found. Although not externally validated, this bacterial signature was found to have a remarkable ability to distinguish GC from non-malignant gastric diseases with a high accuracy (AUC of 91%) [85]. These findings underscore the potential of using the salivary microbiota as a non-invasive biomarker for gastric malignancy. In addition, other studies emphasize the potential utility of the salivary microbiota—particularly the relative abundance of certain oral-derived bacteria—as a non-invasive screening tool for GC, reaching predictive accuracies as high as 91% [85].

Additionally, alterations in stomach-associated microbial communities have been linked to histological stages of gastric carcinogenesis, revealing that certain bacterial genera, such as *Veillonella* and *Enterobacteriaceae*, may be enriched in cancerous tissues compared to normal gastric mucosa [36,86]. For instance, Kwon et al. demonstrated that the transplantation of the human gastric microbiota into germ-free mice recapitulated premalignant changes, further implying the microflora’s active role in tumorigenesis [87]. Likewise, Liu et al. mapped key differences across various gastric microhabitats in GC patients, revealing specialized microbial niches linked to the cancer stage and immune modulation [77].

### 4.4. Microbiota Modulation in Gastric Cancer

HP eradication is crucial for preventing gastric cancer, and meta-analyses along with randomized trials suggest that a successful eradication reduces the risk by approximately 33–47%, especially in areas with a high HP prevalence, such as East Asia [88]. Nonetheless, up to 30–40% of patients may still face progression to malignancy if notable atrophy, metaplasia, or other advanced precancerous lesions are already established prior to bacterial clearance.

PPIs are commonly employed in eradication protocols because their potent acid suppression enhances antibiotic stability. However, large observational studies have raised concerns that prolonged or intensive PPI therapy may negatively affect gastric physiology, particularly in individuals with a history of HP infections [89,90]. In one retrospective cohort of more than 63,000 HP-eradicated individuals, extended PPI use (exceeding 12 months) was linked to a two- to eightfold increase in the subsequent incidence of gastric cancer [91].

Probiotic supplementation has been suggested both to augment HP eradication rates by 8–10% and to support gastrointestinal health following treatment [92]. A recent meta-analysis [93] including 140 studies on adults and a total of 20,215 patients evaluated the role of probiotics in addition to classic eradication therapies of different durations and lines. The supplementation of probiotics led to higher eradication rates compared to the control group, 84.1 vs. 70.5, respectively (Risk Ratio (RR) 1.17, 95% CI 1.15–1.18); the authors additionally reported that *Lactobacillus acidopilus*, *Saccharomyces boulardii*, *Lactobacillus* + *Bifidobacterium* + *Enterococcus*, and *Lactobacillus* + *Bifidobacterium* + *Bacillus cereus* showed a higher efficacy in increasing eradication rates when added to a 10-day triple therapy, whereas only the first three mentioned probiotic mixtures showed a benefit in a 14-day triple therapy.

Beyond the application of probiotics in HP eradication, their use has recently been tested in different steps of GC treatment. For example, in patients with advanced GC undergoing gastric surgery, it was found that the oral administration of *Clostridium butyricum* could decrease postoperative inflammation [94]. Moreover, in a recent phase II trial, the microbiota from healthy obese donors was transplanted to cachectic patients with metastatic GC prior to chemotherapy, improving the response and overall survival [95]. Additional evidence in the context of chemotherapy shows that butyrate-producing bacteria increase the efficacy of oxaliplatin by activating the IL-12 signaling pathway in both mice and humans [95]. A further possible field of application of microbiota modulation in GC is the use of probiotics before or during immunotherapy. Indeed, it has been reported that *H. pylori* can elude the immune response by inducing the up-regulation of PD-L1 in gastric epithelial cells, which in turn can cause the apoptosis of T cells. However, a negative correlation between responses to immunotherapy and *H. pylori* infections has been highlighted, showing a microbiota-related response to therapy [96]. The role of non-*H. pylori* gastric microbiota modulation in the context of immunotherapy is a field of great interest that will have a fundamental role in future research.

In summary, *H. pylori* eradication significantly reduces GC risk, even though patients with existing advanced precancerous lesions may still develop cancer post-eradication. Preliminary data showed that long-term proton pump inhibitors may increase the gastric cancer risk, while probiotics can enhance HP. Beyond eradication, microbial modulations have shown potential in various steps of GC treatment. However, no sufficient data are currently available to suggest a definite role for microbial modulation in gastric cancer treatment.

## 5. Conclusions

The gut microbiota significantly impacts the development and progression of esophageal and gastric cancers. Microbial imbalance contributes to carcinogenesis through mechanisms such as inflammation, immune modulation, and direct DNA damage. In the context of esophageal cancer, different microbial compositions have been found in all the steps of the cancer cascade, from Barrett’s esophagus to esophageal adenocarcinoma, which are characterized by the relative increase in Gram-negative bacteria in the first phases followed by the enrichment of lactic acid producers. Specific microbial signatures have also been found in esophageal squamous cell carcinoma, with a prominent role of *Porphyromonas gingivalis*, *Fusobacterium nucleatum*, and *Streptococci*. Emerging evidence highlights the role of non-HP bacteria in GC carcinogenesis, with a specific enrichment of nitrate-reducing bacteria other than *Propionibacterium acnes*, *Fusobacterium nucleatum*, *Lactobacilli*, and *Streptococci*. Further research is needed to elucidate the interactions between the gut microbiota and upper gastrointestinal cancers, with the aim of improving the early detection, prognosis, and treatment of upper GI cancers.

## Data Availability

The data presented in this study are openly available in Medline and Embase.
